# A macroepigenetic approach to identify factors responsible for the autism epidemic in the United States

**DOI:** 10.1186/1868-7083-4-6

**Published:** 2012-04-10

**Authors:** Renee Dufault, Walter J Lukiw, Raquel Crider, Roseanne Schnoll, David Wallinga, Richard Deth

**Affiliations:** 1 Food Ingredient and Health Research Institute, Ocean View, HI, USA; 2 United Tribes Technical College, Bismarck, ND, USA; 3 Department of Neuroscience and Ophthalmology, Louisiana State University Neuroscience Center, Louisiana State University Health Sciences Center, New Orleans, LA, USA; 4 Shepherd University, Shepherdstown, WV, USA; 5 Department of Health and Nutrition Sciences, Brooklyn College of City, University of New York, Brooklyn, NY, USA; 6 Institute for Agriculture and Trade Policy, Minneapolis, MN, USA; 7 Department of Pharmaceutical Sciences, Northeastern University, Boston, MA, USA

**Keywords:** Autism, DNA methylation, Environmental epigenetics, Heavy metals, HFCS, PON1, SAM, Zn

## Abstract

The number of children ages 6 to 21 in the United States receiving special education services under the autism disability category increased 91% between 2005 to 2010 while the number of children receiving special education services overall declined by 5%. The demand for special education services continues to rise in disability categories associated with pervasive developmental disorders. Neurodevelopment can be adversely impacted when gene expression is altered by dietary transcription factors, such as zinc insufficiency or deficiency, or by exposure to toxic substances found in our environment, such as mercury or organophosphate pesticides. Gene expression patterns differ geographically between populations and within populations. Gene variants of paraoxonase-1 are associated with autism in North America, but not in Italy, indicating regional specificity in gene-environment interactions. In the current review, we utilize a novel macroepigenetic approach to compare variations in diet and toxic substance exposure between these two geographical populations to determine the likely factors responsible for the autism epidemic in the United States.

## Introduction to macroepigenetics with autism as a case study

Autism is a developmental disorder defined by the American Psychiatric Association (APA) in the Diagnostic and Statistical Manual of Mental Disorders (DSM). The condition is considered a pervasive developmental disorder (PDD) that appears in the first three years of life and affects brain development impacting social and communication skills. Autism is defined by a common set of behaviors, including, but not limited to, observed deficits in nonverbal and verbal communication, lack of social reciprocity, and failure to develop and maintain appropriate peer relationships [1]. Recent estimates suggest that 31% of children with Autism Spectrum Disorder (ASD) also meet diagnostic criteria for Attention-Deficit/Hyperactivity Disorder (ADHD) and another 24% of children with ASD exhibit sub-threshold clinical symptoms for ADHD [2]. The number of children affected by this debilitating disorder remains unknown. As part of this review, we analyze the current United States (U. S.) Department of Education Special Education data to estimate the increase in autism prevalence from 2005 to 2010.

The cause(s) of autism also remain(s) unknown. D'Amelio *et al. *found paraoxonase-1 (PON1) gene variants associated with autism in subgroups of the U. S. population but not in Italy [3]. They attributed the gene variation to greater household use of organophosphate (OP) pesticides in the U.S. compared to Italy. We think a more plausible explanation may lie in the U. S. food supply. As part of this investigation, we also reviewed and analyzed the U.S. Department of Agriculture (USDA) Food Availability Spreadsheets to identify which foods are most frequently consumed by Americans and of those which most frequently contain OP pesticide residue as reported by the U.S. Pesticide Data Program.

During this investigation, we conducted a literature review of all studies published on autism since we published our first Mercury Toxicity Model [4], which explains how mercury exposure, nutritional deficiencies and metabolic disruptions contribute to the development of autism. We evaluated all of the relevant studies and expanded our Mercury Toxicity Model. We then used the expanded model to compare the U.S. and Italian populations to determine what, if any, factors could explain the difference in PON1 gene variation and autism prevalence between the two countries. We propose the term "macroepigenetics" to describe the process of examining food supplies and their impact on body metabolism and gene function along with what is known about environmental exposures across populations.

In studying the larger factors outside the gene and human body that impact gene expression, we can better explain some of the gene-environment interactions that create disease conditions such as autism. There is agreement among many in the psychiatry profession that gene-environment interaction research is essential to understanding the etiology of autism and the other pervasive developmental disorders found in the ASD category [5]. How the scientific community arrives at this understanding is key to solving the problem of rising autism prevalence. By demonstrating the macroepigenetic approach to determine the factors likely responsible for the autism prevalence in the U.S., we hope more scientists will follow our interdisciplinary lead and use macroepigenetics as a research strategy.

## Current U. S. autism prevalence and special education trends

Before the 1980s the prevalence of autism in the U. S. was about 0.05% [6]. In 2006, the Center for Disease Control and Prevention (CDC) reported that the estimated prevalence of autism had increased to between 0.6 and 0.7% of all children [7]. Many scientists and parents believe the autism prevalence rate in the U.S. is much higher than these CDC statistics indicate. U.S. government scientists and collaborators published an article in 2007 indicating that 1.1% of U.S. children aged 3 to 17 years were currently diagnosed with ASD [8].

Special education data have been used in the past to estimate autism prevalence trends in the U.S. [9]. In birth cohorts from 1975 to 1995, increases in autism were greatest for annual cohorts born from 1987 to 1992 [9]. From 1992 to 1995, the autism prevalence increased with each successive year but the increases did not appear as great [9]. Our review of the current special education data indicates the number of children ages 6 to 21 receiving special education services under the Autism category has increased 91% from 2005 to 2010. The number of children in the Developmental Delay category has increased 38% and the number of children receiving special education and related services under the Other Health Impaired (OHI) category has increased 26% from 2005 to 2010. Children with a diagnosis of ADHD are included in the OHI category. These increases are startling given that the overall number of children receiving special education services decreased by 5% from 2005 to 2010. Table 1 provides a graphical representation of the data obtained from the Data Accountability Center and analyzed during this review [10].

**Table 1:** Number of U.S. students ages 6 to 21 receiving special education services by disability category and year

Year	Autism	OHI	ED	Speech/Language	Developmental Delay (3 to 9 yrs only)	All Disabilities
2005	193,637	561,028	472,384	1,157,215	79,070	6,109,569

2006	224,594	599,494	458,881	1,160,904	89,931	6,081,890

2007	258,305	631,188	440,202	1,154,165	88,629	6,007,832

2008	292,818	648,398	418,068	1,121,961	96,923	5,889,849

2009	333,234	678,970	405,475	1,107,428	104,528	5,882,157

2010	370,011	704,250	387,556	1,090,378	109,121	5,822,808

**% change (2005-2010)**	**+91%**	**+ 26%**	- 18%	- 6%	**+ 38%**	**- 5%**

%, percent; +, Increased; -, Decreased; ED, Emotional Disturbance; OHI, Other Health Impaired

Data from the 1997 to 2008 National Health Interview Surveys conducted by the CDC confirm these findings of increasing prevalence in autism and developmental disabilities associated with or sharing the diagnostic criteria for autism [11]. Regardless of the source of data it seems clear that autism prevalence is rising in the U.S. compared to other countries, such as Italy, where the autism prevalence in the general population is estimated at only 0.1% [12]. Because autism prevalence rates vary by country, population and geographic location, it is becoming more evident that gene-environment interactions are at play with dietary factors. The influence of environment factors on gene expression is primarily mediated by epigenetic mechanisms, including deoxyribonucleic acid (DNA) methylation along with methylation, acetylation, ubiquitination and phosphorylation of histones. Epigenetic regulation is particularly important during neurodevelopment [13].

## A macroepigenetic model to explain gene-environment interactions in autism

In public health, epidemiology arguably has led the way in researching gene-environment interactions by studying how genotypes, environmental exposures and disorder outcomes occur in the human population [5]. However, this epidemiological approach has often resulted in contradictory scientific conclusions when its practitioners do not consider the dietary factors that interact and modulate the molecular and genetic mechanisms underlying human metabolism and brain function [14]. This has been the case despite the existence of literature from the field of "nutrigenomics", which has specifically studied the effects of food and food ingredients on gene expression. In identifying the public health and the social and/or environmental determinants of disease, it seems invalid to study epidemiology without nutrigenomics, or vice versa. In other words, a more macro-level approach to unraveling the full range of environmental and genetic factors contributing to these kinds of neurological disorders ought to include nutrition factors as a component of the environment. By combining information derived from both nutrigenomic and epidemiology studies, a macroepigenetic model has already been developed to explain some of the gene-environment interactions with dietary factors that lead to the development of autism and ADHD [4].

**Figure 1:**
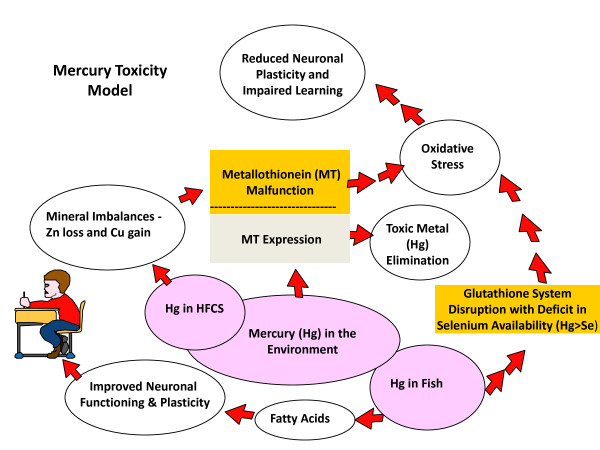
**The original Mercury Toxicity Model**. The original Mercury Toxicity Model was published in 2009 by Dufault *et al. *in the *Behavioral and Brain Functions *journal. The model is a flow chart of what can happen in the body when there is exposure to mercury (Hg) from ingestion of foods (via HFCS, food colors and fish) or inhalation of air. Human neurodevelopment can be adversely impacted when MT gene expression is altered or suppressed by dietary transcription factors such as zinc (Zn) insufficiency or deficiency. Without proper MT expression and function, mercury excretion may not be possible and oxidative stress in the brain from mercury insult leads to reduced neuronal plasticity and impaired learning. Hg in fish is a problem when there is not enough selenium (Se) in the fish to counteract the Hg and the glutathione (GSH) system is disrupted leading to further oxidative stress.

Figure 1 shows the Mercury Toxicity model that provides a macroepigenetic explanation of how human neurodevelopment can be adversely impacted when gene expression is altered by dietary transcription factors such as zinc insufficiency or deficiency, or by exposure to toxic substances found in our environment, such as the heavy metals mercury and copper [4]. Elimination of heavy metals requires the expression of the metallothionein (MT) gene, which synthesizes the Zn-dependent metal binding protein metallothionein [15]. With dietary zinc (Zn) loss and copper (Cu) gain from the consumption of high fructose corn syrup (HFCS) [16], metabolic processes required to eliminate heavy metals are impaired in children with autism [4]. Mercury has been found in samples of high fructose corn syrup and is allowable in trace amounts in certain food colors so long as the concentration does not exceed one part per million [17,18]. Mercury (Hg) and specific other heavy metals, including lead (Pb), copper (Cu), cadmium (Cd), silver (Ag) and bismuth (Bi), are capable of displacing the Zn atom in the MT protein molecule [15]. This 'pathogenic' displacement of Zn impairs the MT molecule and its ability to pick up the heavy metal and carry it out of the body. If the diet is deficient in Zn or the absorption of Zn is impaired, then the body may not produce enough MT protein to carry and excrete the heavy metal load [19,20]. Children with autism may be Zn deficient and often have MT dysfunction [21-23]. Because of their diminished capacity to excrete toxic heavy metals, the severity of their condition is associated with their toxic metal burden [24]. This macroepigenetic model proposes that autism prevalence is related to the consumption of HFCS and the overall exposure to Hg in the U.S. [4]. However, other dietary factors associated with the consumption of HFCS may further contribute to the development of autism in the U.S.

## Additional dietary factors associated with consumption of HFCS

U.S. per capita consumption of HFCS in 2009 was 35.7 pounds per year [25]. The peak years for annual consumption of HFCS coincided with the peak growth rates of ASD in California, the only state that reports number of cases of ASD dating back to the mid-1980s [4]. The Mercury Toxicity Model shows the HFCS characteristics most likely contributing to autism include the zinc-depleting effect that comes from consuming HFCS and certain food colors found in processed foods, and the additional Hg exposure that may occur from the low Hg concentrations sometimes found in HFCS as a result of the manufacturing process [4,17]. This model can be expanded to include additional adverse effects associated with the consumption of HFCS that likely contribute to the development of autism through PON1 gene modulation and lead intoxication.

U.S. Department of Agriculture (USDA) scientists warn that when dietary intake of magnesium (Mg) is low, consumption of HFCS leads to lower calcium (Ca) and phosphorus (P) balances adversely affecting macromineral homeostasis in humans [26]. This is an unfortunate finding because there is evidence to suggest that dietary intake of Mg is low among Americans, most of whom consume a high fructose diet. In 2003, CDC scientists reported that substantial numbers of U.S. adults fail to consume adequate Mg in their diets [27]. Children with autism were found to have significantly lower plasma Mg concentrations than normal subjects [28]. Adams *et al. *found significant reductions in red blood cell (RBC) Ca, serum and white blood cell (WBC) Mg and an increase in RBC copper in autistic children as compared to controls [29]. Recently, USDA scientists reported that the National Health and Nutrition Examination Survey (NHANES) data for 2005 to 2006 indicate that overall, nearly one half of all individuals one year and over had inadequate intakes of dietary Mg [30]. With a substantial number of Americans consuming inadequate amounts of dietary Mg along with HFCS diets, one may predict that substantial numbers of Americans are likely experiencing a calcium (Ca) deficit as well.

Insufficient intake of dietary Ca, Mg and Zn, or losses or displacement of any of these minerals from the consumption of HFCS, may further enhance the toxic effects of lead (Pb) on cognitive and behavioral development in children [31]. A significant and independent inverse relationship between dietary Ca intake and blood Pb concentrations was found in 3,000 American children examined as part of NHANES II [32]. Elevated blood Pb levels are indicative of Pb intoxication, which is found in some children diagnosed with autism and associated with the development of ADHD [33,34]. It may be that inadequate intake of Ca or Mg combined with a HFCS zinc-depleting diet increases the risk of developing autism and ADHD from Pb intoxication.

**Figure 2:**
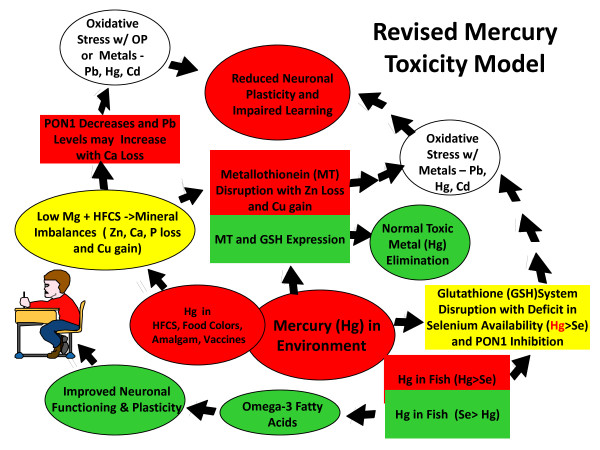
**The expanded Mercury Toxicity Model**. Figure 2 shows the expanding Mercury Toxicity Model that includes changes both in lead (Pb) toxicity and human serum paraoxonase (PON1) activity when dietary intake of Mg is low and consumption of high fructose corn syrup (HFCS) leads to lower calcium (Ca) and phosphorus (P) balances, adversely affecting macromineral homeostasis. With insufficient dietary intake of Ca and/or Mg, children become more susceptible to Pb intoxication and OP exposures with decreasing PON1 activity. Pb intoxication and OP exposures can both lead to oxidative stress in the brain reducing neuronal plasticity.

Inadequate intake of Ca or Mg may further contribute to these developmental disorders by impacting human serum paraoxonase-1 (PON1) gene expression. PON1 is a calcium dependent enzyme responsible for OP pesticide detoxification as well as hydrolysis of the thiolactone form of homocysteine [35,36]. PON1 is synthesized in the liver and secreted in blood where it is incorporated into high density lipoproteins (HDL). The availability and catalytic activity of PON1 are impaired in many children with ASD making them more susceptible to the toxic effects of OP pesticide residues which are most frequently found in grain [37,38]. Figure 2 shows the expanded Mercury Toxicity Model that includes changes both in Pb toxicity and PON1 activity when dietary intake of Mg is low and consumption of HFCS leads to *greater loss of calcium *(Ca) and phosphorus (P), thereby adversely affecting macromineral homeostasis.

## PON1 activity and organophosphate exposure in U.S

One can assert that with the consumption of a HFCS intensive diet and inadequate Mg intakes, PON1 activity may decrease, along with resulting Ca losses in genetically predisposed individuals. Although there are no human data yet to support this assertion, PON1 activity in rats decreased when fed a HFCS diet to mimic the human metabolic syndrome [39]. PON1 activity has been extensively studied in humans and there are a number of factors known to modulate or alter PON1 expression including, but not limited to, Hg exposure, sex and age [40,41]. Age plays the most relevant role, as PON1 activity is very low before birth and gradually increases during the first few years of life in humans [41]. In one study, scientists at UC Berkeley found the PON1 levels in many children may remain lower than those of their mothers for several years, especially those with genotypes associated with decreased PON1 activities [42]. The scientists concluded that these children may be more susceptible to OP pesticides throughout their childhood and more vulnerable to conditions associated with oxidative stress such as autism [42]. In a different study, scientists at UC Berkeley found that two-year-old children were less likely to display symptoms of PDD when their mothers had higher paraoxonase levels during their pregnancy [43]. Proper function and adequate expression of the PON1 gene is essential both for prenatal development and child health because exposure to OP pesticides is a common occurrence in the U.S.

The CDC tracks exposure to OP pesticides or their metabolites through the National Biomonitoring Program (NBP). Exposure data are reported for the population as a whole and for subgroups. While most American groups are exposed to OP pesticides, children ages 6 to 11 have significantly higher exposures than adults and are at greatest risk from OP neurotoxicity [44]. Harvard University researchers recently reported finding OP pesticide residues in a number of foods frequently consumed by children [45]. The researchers expressed concern that the children were at times being exposed to multiple pesticide residues in single food commodities. OP pesticide exposures occur primarily from the consumption of foods containing pesticide residues.

It is well known that pesticide exposure can impair neurodevelopment in children, but recent studies have found that pesticide exposure during pregnancy can also cause delayed mental development in children [46]. A review of epidemiological studies in 2008 found that prenatal and childhood exposure to OPs impairs neurobehavioral development [47]. Higher concentrations of urinary dialkyl phosphate (DAP) measured during pregnancy was significantly associated with lower cognitive scores in children at seven years of age. Those children in the highest quintile of maternal DAP concentrations had an IQ score seven points lower than those children in the lowest quintile [48]. In a group of newborns with the highest levels of the organophosphate chlorpyrifos measurable in their umbilical cord blood, birth weight averaged 150 grams less than the group with the lowest exposure [49]. Prenatal pesticide exposure showed deficits consistent with developmental delays of 1.5 to 2 years [49].

Diet is the main source of OP exposure in children. Under the 1996 Food Quality Protection Act, the U.S. Secretary of Agriculture is directed to collect pesticide residue data on commodities frequently consumed by infants and children. USDA Pesticide Data Program (PDP) provides the residue data to comply with this law [50]. We reviewed the PDP data from 2004 to 2008 and identified the foods most frequently found to contain organophosphate insecticide residues. In addition, we obtained the per capita availability data from the USDA to determine the amount of each food commodity the average American consumes [25]. The results of our review indicate that wheat and corn are the commodities most likely contributing to OP exposure in U. S. children. Estimated per capita wheat consumption was approximately 95 pounds per year while estimated per capita corn consumption was approximately 23 pounds per year. The primary use of corn is for the production of corn sweeteners, such as HFCS; however, pesticide residue data were not gathered for this commodity by the PDP. Table 2 provides a complete breakdown of the results of this data review.

**Table 2:** PDP residue detections by year sampled wi th U.S. per capita consumption data

Year	Crop	U.S. Per Capita Avail. (lbs.)	OP Residue Detected	% Samples w/Detects
2004	wheat	**94.8**	Chlorpyrifos methyl	**20.8**

2004	wheat	**94.8**	Malathion	**49.4**

2005	wheat	**94.6**	Chlorpyrifos methyl	**23.1**

2005	wheat	**94.6**	Malathion	**66.9**

2006	wheat	**95.6**	Chlorpyrifos methyl	**16.7**

2006	wheat	**95.6**	Malathion	**63.0**

2007	corn	22.8*	Chlorpyrifos	**30**

2007	corn	22.8*	Malathion	**37.9**

2007	corn	22.8*	Pirimiphos methyl	**2.4**

2007	celery	3.79	Dimethoate	10.8

2007	celery	3.79	Omethoate	16.5

2007	celery	3.79	Malathion	21.2

2007	peaches	2.168	Chlorpyrifos	18

2007	peaches	2.168	Phosmet	36.2

2007	almonds	1.1	Chlorpyrifos	46

2007	almonds	1.1	Phosmet	4.4

2007	almonds	1.1	Dichlorvos	0.6

2007	fresh blueberries	0.384	Phosmet	9.6

2007	frozen blueberries	1.392	Phosmet	36.4

2007	fresh blueberries	0.384	Chlorpyrifos	1.3

2007	frozen blueberries	1.392	Chlorpyrifos	4.5

2007	fresh blueberries	0.384	Malathion	4.9

2007	frozen blueberries	1.392	Malathion	4.5

2008	corn	23.2*	Chloropyrifos	**17.8**

2008	corn	23.2*	Malathion	**33.7**

2008	apple juice	15.93	Phosmet	1.9

2008	rice	14.8	Malathion	4.3

2008	strawberries	3.965	Malathion	24.6

2008	celery	3.79	Dimethoate	9.3

2008	celery	3.79	Omethoate	17.4

2008	celery	3.79	Malathion	19.3

2008	peaches	2.462	Chlorpyrifos	17.2

2008	peaches	2.462	Phosmet	30.7

2008	almonds	1.1	Chlorpyrifos	35.5

2008	almonds	1.1	Dichlorvos	4.3

2008	almonds	1.1	Phosmet	5.9

2008	fresh blueberries	0.526	Phosmet	11.6

2008	frozen blueberries	1.447	Phosmet	22.2

2008	fresh blueberries	0.526	Chlorpyrifos	1.7

2008	frozen blueberries	1.447	Chlorpyrifos	5.6

2008	fresh blueberries	0.526	Malathion	4.4

2008	frozen blueberries	1.447	Malathion	27.8

%, Percent; Avail., Availability; lbs, Pounds; OP, Organophosphate; PDP, Pesticide Data Program; U.S., United States; w, with; * Corn grain only, corn sweeteners not included

From Table 2 it is clear consumers are at risk of exposure to multiple OP pesticide residues from consuming the very same commodity. Cumulative exposures will continue to occur in the U.S. where OP pesticide use is widespread by the agricultural industry. Although OP pesticide use is equally widespread in other countries, there is genetic variation across populations that determine degree of susceptibility to OP exposure. The PON1 gene variants associated with autism in subgroups of the U.S. population but not in Italy could be attributed to the fact that HFCS consumption rarely occurs in Italy, thereby lessening the conditions for PON1 modulation.

## HFCS consumption and PON1 modulation in autism in the U.S

**Figure 3:**
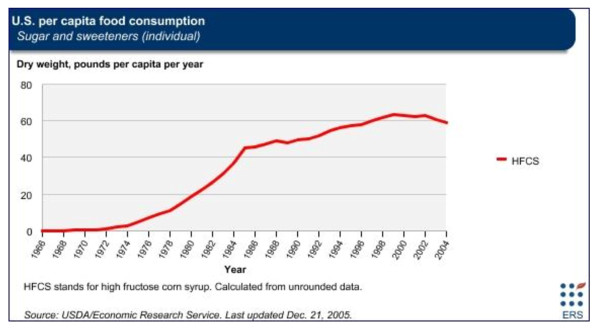
**U.S. per capita consumption of high fructose corn syrup 1966-2004**. Figure 3 shows the United States (US) per capita consumption of high fructose corn syrup (HFCS) in pounds per year as calculated by the United States Department of Agriculture (USDA)/Economic Research Service.

In the 27-member European Union (EU), of which Italy is an original participant, HFCS is known as "isoglucose" and currently it is rarely consumed by Italians. Americans on the other hand consume on average 35.7 pounds per year, which may increase their overall Hg exposure [17,25]. Figure 3 shows U.S. per capita food consumption in pounds per year for HFCS beginning in the early 1970s and increasing throughout the 1980s to reach a peak between 1999 and 2002. In our previous publication, we reported the peak years for annual consumption of HFCS in the U.S. occurred within the same period as when the annual growth rates of autism peaked in California [4].

American per capita consumption of HFCS has exceeded 20 pounds per year since 1980 while Italians consume negligible amounts of the same ingredient. As was previously mentioned, mercury (Hg) and fructose may both modulate PON1 activity [39-41]. While excessive fructose exposure in the U.S. may primarily occur through the consumption of foods containing HFCS, mercury exposure may occur in a number of ways. A comparison of common sources of mercury exposure in the U.S. and Italy may offer a further explanation of the PON1 gene variation associated with autism in the U.S. but not in Italy.

In addition to HFCS, primary sources of inorganic and elemental Hg exposure may occur from consumption of food colors and preservatives made with mercury-cell chlorine or chlor-alkali products, seafood consumption, Hg in dental amalgam, thimerosal in vaccines, and depending on geographic location, inhalation of Hg contaminated air [4,51-54]. Children living near coal-fired power plants are often exposed to higher levels of Hg in their breathing air and have a higher prevalence of autism [55]. Because Hg emissions from coal-fired power plants are not yet regulated in either the U.S. or Italy, this particular source of Hg exposure is unlikely to explain the overall difference in autism prevalence between these two countries. With respect to the consumption of seafood, use of Hg dental amalgam, thimerosal in vaccines or Hg-containing food colors and preservatives, there is also no appreciable difference between Italy and the U.S. [56-58]. The only remaining variable in our model is the excessive consumption of HFCS by Americans, which results in greater chronic exposures to both inorganic Hg and, by definition, fructose [4].

Inorganic Hg may interact with cysteine residues on PON1 preventing its activation in the liver and impairing the body's ability to protect itself against OP pesticides and oxidative stressors involved in autism [41]. As noted above, PON1 is responsible for hydrolysis of homocysteine thiolactone, and plasma PON1 activity is negatively correlated with homocysteine levels [36,59]. Homocysteine is a metabolic biomarker for oxidative stress and impaired methylation capacity. A recent study of the Inuit population found a significant inverse correlation between PON1 activity and Hg levels, as well as a direct correlation with selenium levels [60]. With increasing Hg and fructose exposure and reductions in dietary Ca, one can expect to see reduced PON1 activity and increasing homocysteine levels in children with ASD.

Indeed, Pasca *et al. *recently reported finding that both PON1 arylesterase and PON1 paraoxonase activities were decreased in children with autism [61,62]. James *et al. *found that children with autism had higher plasma homocysteine levels than controls but demonstrated significant improvements in transmethylation metabolites and glutathione (GSH) after receiving folate and vitamin B12 [63]. Patel and Curtis found that in addition to glutathione and B12 injections one to three times per week, children with autism and ADHD showed significant improvement in many areas of social interaction, concentration, writing, language and behavior when fed an organic diet low in fructose and free of food additives and food colors [64].

Mothers of autistic children in the U.S. were also found to have significant increases in mean plasma homocysteine levels compared to controls [65]. Schmidt *et al. *found that women who took vitamin supplements during the periconceptional period reduced the risk of autism in their children [66]. Those women who did not take vitamins during this period were more likely to have a child with autism and were at even greater risk when they had specific genetic variants within one-carbon metabolism pathways. This suggests that folate and other dietary methyl donors may alter epigenetic regulation of gene expression in their children, thereby reducing the risk of autism [66].

## Methionine synthase links oxidation to epigenetics

Epigenetic regulation of gene expression is highly dependent upon methylation of both DNA and histones, and methylation capacity is in turn dependent upon activity of the folate and vitamin B12-dependent enzyme methionine synthase, which converts homocysteine to methionine. Lower methionine synthase activity decreases the level of the methyl donor S-adenosylmethionine (SAM) while simultaneously increasing the level of the methylation inhibitor S-adenosylhomocysteine (SAH) [67]. The combined effect of changes in the SAM to SAH ratio, therefore, exerts a powerful influence over more than 200 methylation reactions, including DNA and histone methylation [68].

**Figure 4:**
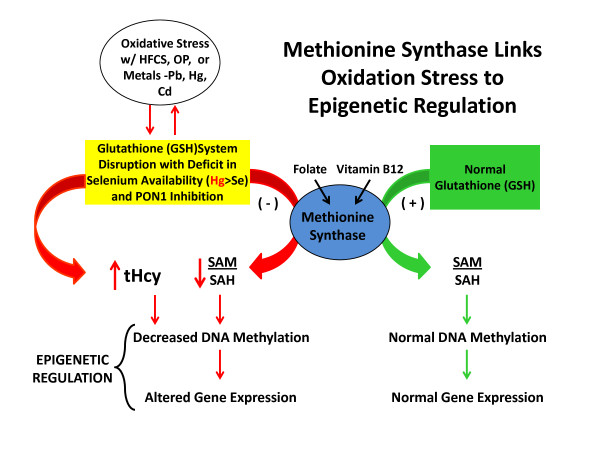
**Methionine synthase links oxidative stress to epigenetic regulation**. Figure 4 shows how exposure to toxic substances, such as OP pesticides, HFCS, or heavy metals, inhibits methionine synthase through effects of oxidative stress. As a result, decrease of SAM to SAH ratio will lead to a decrease in DNA methylation and consequently to altered PON1 gene expression.

Methionine synthase activity is inhibited by oxidative stress, and its inhibition results in the diversion of homocysteine to produce the antioxidant glutathione (GSH), providing an important adaptive response [69]. However, oxidative inhibition of methionine synthase leads to epigenetic effects via the resultant decrease in the SAM to SAH ratio and decreased DNA and histone methylation. Epigenetic changes in gene expression can recruit further adaptive responses to oxidative stress. Figure 4 illustrates how these changes may occur when the body is under oxidative stress from exposure to OP pesticides, heavy metals, and calcium depleting substances, such as HFCS. Decreased methionine synthase activity during oxidative stress also increases homocysteine thiolactone formation [70], raising the importance of PON1. As was previously mentioned, PON1 is essential for reducing homocysteine levels, which are thought to be harmful. Elevated plasma homocysteine (tHcy) levels are associated with genome-wide DNA hypomethylation that may carry over from one generation to the next, increasing the risk of autism [71]. Epigenetic changes affecting germline cells can give rise to these transgenerational effects [72]. James *et al. *found that parents share similar metabolic deficits in methylation capacity and glutathione-dependent antioxidant/detoxification capacity with their children with autism [71].

## Synergistic effect of multiple neurotoxins

Based upon the discussion above, it is clear that methionine synthase activity is crucial for translating changes in oxidative status into epigenetic effects, and this role is confirmed by the improved metabolic profile in autistic subjects given folate and vitamin B12 [63]. This relationship has given rise to the "Redox/Methylation Hypothesis of Autism", which proposed that oxidative insults arising from environmental exposures, such as Hg and pesticides, can cause neurodevelopmental disorders by disrupting epigenetic regulation [73]. The macroepigenetic Mercury Toxicity Model expanded in this paper provides additional support for the "Redox/Methylation Hypothesis of Autism" while contributing important insight into the oxidative stress feedback mechanisms that may occur as a result of malnutrition resulting from dietary exposures to toxins. The delivery of children exhibiting autistic behaviors might be associated with the prenatal diet of their mothers. The severity of these behaviors can be further exacerbated by toxic dietary exposures of the children, which can improve with dietary changes aimed at eliminating these exposures. Children with autism could well be exhibiting an epigenetic response to several neurotoxic substances at once, including, but not limited to, inorganic Hg, Pb, OP pesticides and/or HFCS. The combined effect of these substances acting together is likely greater than the sum of the effects of the substances acting by themselves. This effect likely reduces neuronal plasticity and impairs learning capacity in autistic children.

## Conclusion

The number of children ages 6 to 21 in the U.S. receiving special education services under the autism disability category increased 91% between 2005 to 2010 despite fewer children receiving special education services overall during the same time period. A comparison of autism prevalence between the U.S. and Italy using the Mercury Toxicity Model suggests the increase in autism in the U.S. is not related to mercury exposure from fish, coal-fired power plants, thimerosal, or dental amalgam but instead to the consumption of HFCS. Consumption of HFCS may lead to mineral imbalances, including Zn, Ca and P loss and Cu gain and is a potential source of inorganic mercury exposure. These mineral imbalances create multiple pathways for oxidative stress in the brain from exposure to OP pesticides and heavy metals, such as Pb or Hg. Inorganic mercury and fructose exposure from HFCS consumption may both modulate PON1 gene expression. With a reduction in PON1 activity, there is a potential for increasing homocysteine levels which are associated with genome-wide DNA hypomethylation that may carry over from one generation to the next, affecting both neurodevelopment and autism prevalence.

## Abbreviations

ADHD: Attention-Deficit-Hyperactivity-Disorder; Ag: Silver; APA: American Psychiatric Association; ASD: Autism Spectrum Disorder; Bi: Bismuth; Ca: Calcium; Cd: Cadmium; CDC: Center for Disease Control and Prevention; Cu: Copper; DAP: Dialkyl phosphate; DSM: Diagnostic and Statistical Manual of Mental Disorders; EU: European Union; GSH: Glutathione; HDL: High density lipoprotein; HFCS: High fructose corn syrup; Hg: Mercury; Mg: Magnesium; MT: Metallothionein; NBP: National Biomonitoring Program; NHANES: National Health and Nutrition Examination Survey; OHI: Other Health Impaired; OP: organophosphate pesticide; P: phosphorus; Pb: lead; PDD: Pervasive Developmental Delay; PDP: Pesticide Data Program; PON1: paraoxonase-1; RBC: red blood cell; SAH: S-adenosylhomocysteine; SAM: S-adenosylmethionine; tHcy: total plasma homocysteine; USDA: United States Department of Agriculture; WBC: White blood cell; Zn: Zinc.

## Competing interests

RDeth has in the past received compensation as an expert witness on the topic of autism. All of the other authors declare that they have no competing interests.

## Authors' contributions

RDufault spearheaded the review and recruited interdisciplinary collaborators to contribute to the development of the manuscript. RDufault was the lead investigator and literature reviewer for the expansion of the mercury toxicity model. RDufault acquired, analyzed and interpreted the new data sets. WJL, RS and RDeth helped revise the manuscript critically for important intellectual content. RDeth provided Figure 4 and was the primary author of the methionine synthase section. All authors read and approved the final manuscript. RS validated all of the references. RC validated the data in Table 1 to ensure it was correctly gathered from existing data bases. RC also double checked the calculations in Table 1 to ensure they were error free. DW helped draft and edit the manuscript.

## Lead author's information

After retiring with honors from her position as an environmental health officer with the U.S. Public Health Service, R. Dufault obtained her teaching license and taught elementary special education for 3.5 years at which time she gained an understanding of how children with autism and ADHD in the U.S. receive special education services. As a volunteer, she currently teaches an epigenetics course on-line through the Food Ingredient and Health Research Institute.
